# Effect of Non-Calcined Sugarcane Bagasse Ash as an Alternative Precursor on the Properties of Alkali-Activated Pastes

**DOI:** 10.3390/molecules27041185

**Published:** 2022-02-10

**Authors:** Leila Nóbrega Sousa, Pâmella Fernandes Figueiredo, Sâmara França, Marcos Vinicio de Moura Solar Silva, Paulo Henrique Ribeiro Borges, Augusto Cesar da Silva Bezerra

**Affiliations:** 1 Federal Center for Technological Education of Minas Gerais, Belo Horizonte 30421-169, Brazil; leilanobrega2@hotmail.com (L.N.S.); pamella-fernandes@hotmail.com (P.F.F.); samara_franca@yahoo.com.br (S.F.); paulo.borges@cefetmg.br (P.H.R.B.); 2 Cemig Geração e Transmissão S.A., Belo Horizonte 30190-924, Brazil; vinicio@cemig.com.br

**Keywords:** sugarcane bagasse ash, alkali-activated paste, alternative precursor, ambient temperature curing

## Abstract

BFS-MK-based alkali-activated materials are well established as an alternative for sustainable and green construction. This work aims to collaborate and encourage the use of biomass ashes, such as sugarcane bagasse ash (SCBA), as a precursor in alkali-activated materials (AAM). This ash is a rich source of aluminosilicate, which is a primary requirement for this application. In addition, this waste is still an environmental liability, especially in developing countries, and with a large volume of annual production. Thus, in this research, alkali-activated pastes (AA) were produced using sugarcane bagasse ash (SCBA), granulated blast furnace slag (BFS) and metakaolin (MK) as precursors. In addition, environmental gains were encouraged with energy savings, with no extra reburn or calcination steps in the SCBA. Thus, the precursors were characterized by laser granulometry, X-ray fluorescence spectrometry (XRF), X-ray diffraction (XRD), scanning electron microscopy (SEM) and Fourier transform infrared spectroscopy (FTIR). The pastes were produced by mixing the precursors with the activator, composed of a mixture of sodium hydroxide 8 mol/L and sodium silicate. Aiming to study the incorporation of SCBA, all samples have a precursor/activator ratio and a BFS/(BFS + MK) ratio constant of 0.6. The compressive strength analysis, FTIR, XRD, TGA, SEM and isothermal calorimetry analyses pointed out the occurrence of alkaline activation in all proposed samples for curing times of 7, 28 and 91 days. The sample GM0.6-BA0 (15% SCBA) achieved the highest compressive strength among the samples proposed (117.7 MPa, at 91 days), along with a good development of strength throughout the curing days. Thus, this work presents the properties of alkaline-activated pastes using SCBA as a sustainable and alternative precursor, seeking to encourage the use of raw materials and alternative waste in civil construction.

## 1. Introduction

The three-dimensional inorganic polymeric chain structure of the alkali-activated material (AAM) is formed from the reaction of a solid aluminosilicate in a strongly alkaline solution [1]. The possibility of applications for this material is wide, such as in paste, mortar and reinforced concrete [2,3]. In general, precursors can be from natural sources, by-products and industrial residues [1]. Blast furnace slag (BFS) and metakaolin (MK) are the most widely used materials as aluminosilicate sources for AAM. BFS is a by-product of the steel industry, and as it is composed of network-forming anions, such as (SiO_4_)^4−^ and (AlO_4_)^5−^, and network-modifying cations, such as Ca^2+^, Al^3+^ and Mg^2+^ [3], its alkaline activation has been extensively studied. Moreover, metakaolin is generated from the calcination of kaolin, being composed mainly of SiO_2_ and Al_2_O_3_, and the process results in a disordering of the structure, and in the release of aluminum and silicon, which makes the MK structure highly reactive. In recent decades, research has been carried out with different wastes and by-products, such as rice husk ash [4,5], red mud [6], iron tailings [7], reclaimed asphalt pavement [8,9], eucalyptus biomass ash [10], sugarcane bagasse ash [11,12], among others. These works are encouraged by the growing search of the civil construction sector to incorporate sustainability into its processes and products. Some changes are already being applied, and the alkali-activated material shows promise as an alternative to Portland cement in several applications [13,14].

Basically, sugarcane bagasse ash is a residue from sugarcane mills, and it is commonly produced in the cogeneration boilers after the use of bagasse as fuel to produce electricity [15]. Its importance lies in the fact that this plant is the raw material for important consumed products, i.e., sugar and alcohol. The joint production of Brazil, India, China and Thailand corresponds to more than 75% of the world sugarcane production; Brazil alone produced more than 752 mil ton in 2019 [16]. However, as for most agricultural residues, the disposal of this ash is a huge issue, as it is still disposed in landfills in most sugar and ethanol mills, causing environmental damage in water, air and land [17]. It is well known that one of the difficulties in the wide reuse of solid waste, such as SCBA, is the variability of the characteristics of these materials due to geographic and processing variability [18]. In general, SCBA contains cellulose, hemicellulose and lignin [18] and research from different countries shows that the main content of inorganic compounds of SCBA are SiO_2_, Al_2_O_3_ and CaO [19,20,21,22]. The conditions under which the bagasse is burned in the boiler (600–800 °C) for electricity generation produces ash with polymorphic and amorphous crystalline phases [21,23,24,25].

Preliminary studies have shown the feasibility of using this ash as a precursor for alkali activation binder [11,12,26,27,28,29,30]. Castaldelli et al. [12] and Pereira et al. [29] studied the partial replacement of granulated blast furnace slag (BFS) by SCBA, while Tippaysam et al. [11] and Castaldelli et al. [28] studied the binary system fly ash and SCBA. Yadav et al. [26] studied the partial replacement of metakaolin (MK) by SCBA and Tchakouté et al. [30] evaluated the utilization of sodium waterglass from SBCA as activator for producing MK-based geopolymer. Recently, Akbar [27] used SBCA as exclusive precursor for geopolymer production reinforced with propylene fibers. As can be seen, all these works used at most a binary mixture for the geopolymers production and, in addition, only Castaldelli et al. [12] used SCBA in the way it was collected at the factory. All other works carried out the reburning of the ash or SCBA calcination under a controlled environment. For that reason, this works aims to evaluated the feasibility of using non-calcined SCBA as a precursor in a ternary alkali-activated material with MK and BFS.

Thus, in this research, the effect of addition of SCBA on the properties of pastes formulated with MK and BFS (BFS:(BFS + MK) ratio of 0.6) was investigated. The paste samples were produced by mixing the precursors with the activator, composed of a mixture of sodium hydroxide 8M and sodium silicate; moreover, SCBA was ground to increase its reactivity, due to the increase in the surface area. Thus, this work aims to collaborate with the studies of SCBA and, therefore, encourage the reuse of biomass ashes, the disposal of which leads to environmental problems, especially in underdevelopment countries.

## 2. Results and Discussion

**Figure 1:**
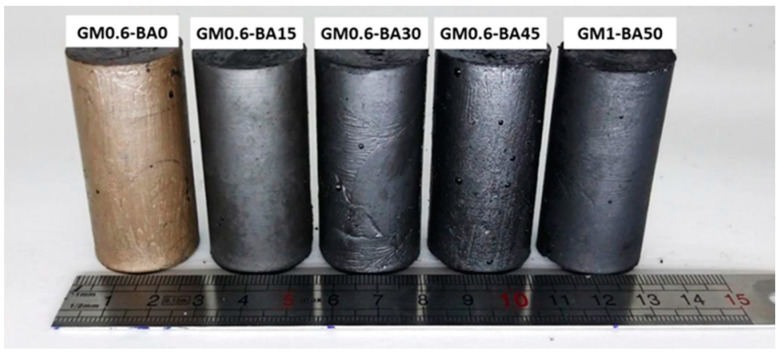
Samples of AA pastes (left to right: GM0.6-BA0, GM0.6-BA15, GM0.6-BA30, GM0.6-BA45 and GM1-BA50).

The appearance of samples presented different colors due to the different amounts of SCBA (Figure 1), ranging from gray-brown in the GM0.6-BA0 sample to black in the other samples. The black color of the samples is due to the presence of SCBA.

**Figure 2:**
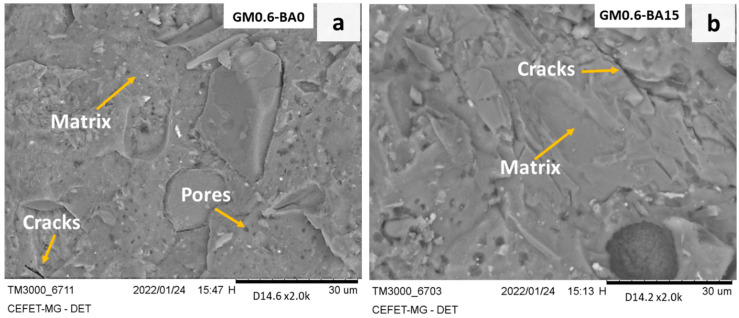

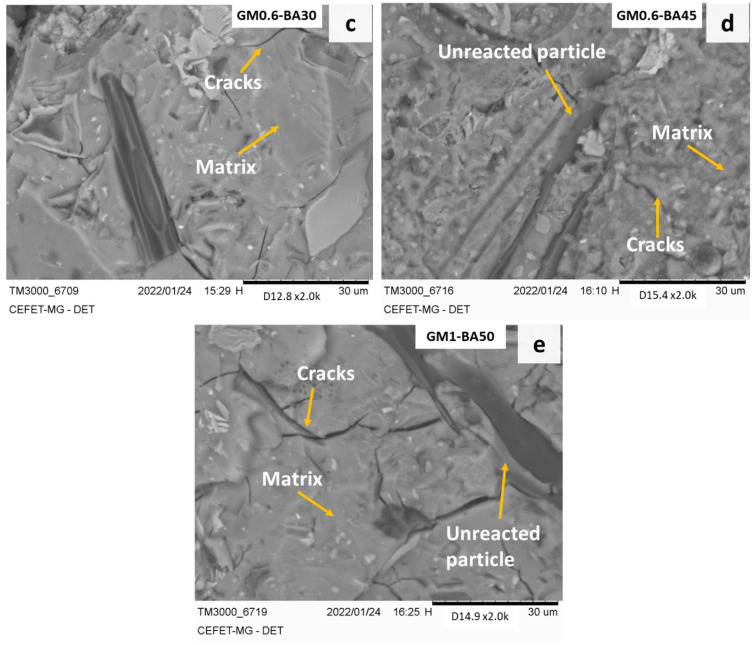
SEM micrograph of AA pastes after 28 days of curing. (**a**) GMA0.6-BA0, (**b**) GMA0.6-BA15, (**c**) GMA0.6-BA30, (**d**) GMA0.6-BA45, (**e**) GMA1-BA50.

Figure 2 illustrates the analysis of micromorphology of alkali-activated pastes after 28 days of curing by SEM. In general, all the samples presented good matrix densification. However, as the analyses were performed after the compressive strength, the presence of cracks was observed in all samples. The GM0.6-BA0 sample (Figure 2a) shows a compact and cohesive matrix, and the presence of a large number of pores. These pores are likely due to the incomplete expulsion of air during the molding process. In samples GM0.6-BA15 and GM0.6-BA30 (Figure 2b,c), a more cohesive microstructure was formed, compared to other samples. In GM0.6-BA45 (Figure 2d) images, the presence of unreacted fibers and particles from the SCBA can be identified. These particles impaired the homogeneous development of the matrix due to a lesser dissolution rate of particles and, consequently, the formation of products, such as CASH-type gel [31]. Comparing the images obtained for samples GM0.6-BA0 to GM0.6-BA45, an increase in the addition of SCBA resulted in a more heterogeneous material. Thus, the presence of unreacted particles, pores and voids probably caused a reduction in strength. Then, it can be assumed that the filling effect occurred by crystalline particles [32,33]. The presence of quartz (silica crystalline phase) in the system is able to contribute to build a cohesive agglutinating phase through the incorporation of this phase in the gel network [34].

**Figure 3:**
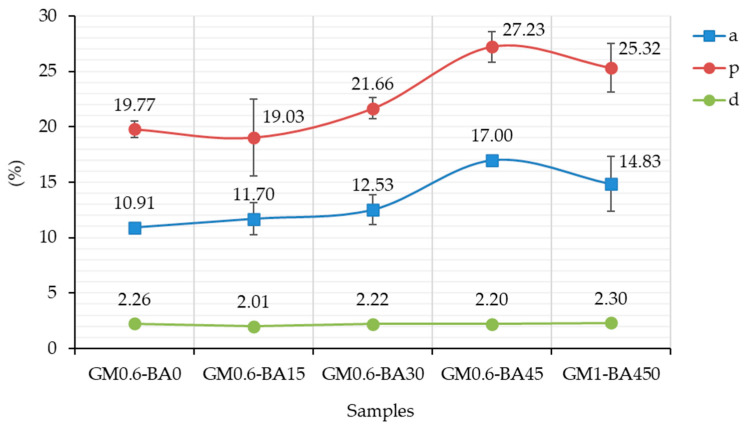
Average values of water absorption (a), apparent porosity (p) and bulk density (d) for samples.

The average values of water absorption (%), apparent porosity and bulk density (%) at 91 days of curing are shown in Figure 3. Comparing samples from GM0.6-BA0 to GM0.6-BA45, the apparent porosity and water absorption enhance as the content of SCBA increase, as also observed by other authors [11,35,36].

**Figure 4:**
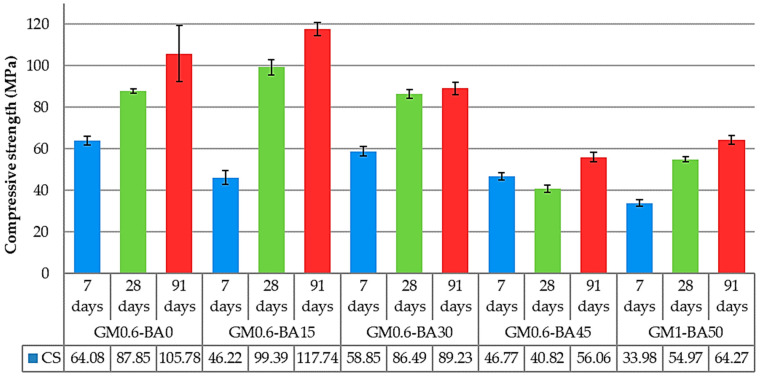
Compressive strength of samples of 7, 28 and 91 days of curing (MPa).

The effect of SiO_2_:Al_2_O_3_ and CaO:SiO_2_ ratios caused by SCBA had an important role in GM0.6-BA015 and GM0.6-BA030 replacements, in order to induce an increase in compressive strengths, and a low water absorption and apparent porosity. However, at higher levels of substitution, GM0.6-BA45 and GM1-BA50, a negative effect stands out, causing a higher water absorption and apparent porosity (Figure 3). This was in accordance with the compressive strength results (Figure 4), suggesting that the samples with major water absorption and apparent porosity also had the lowest compressive strength.

**Figure 5:**
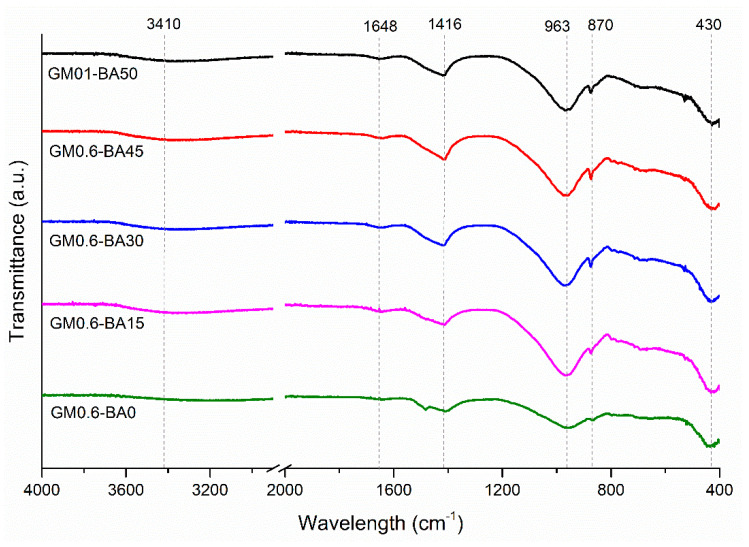
FTIR spectra for pastes after 28 days of curing.

**Figure 6:**
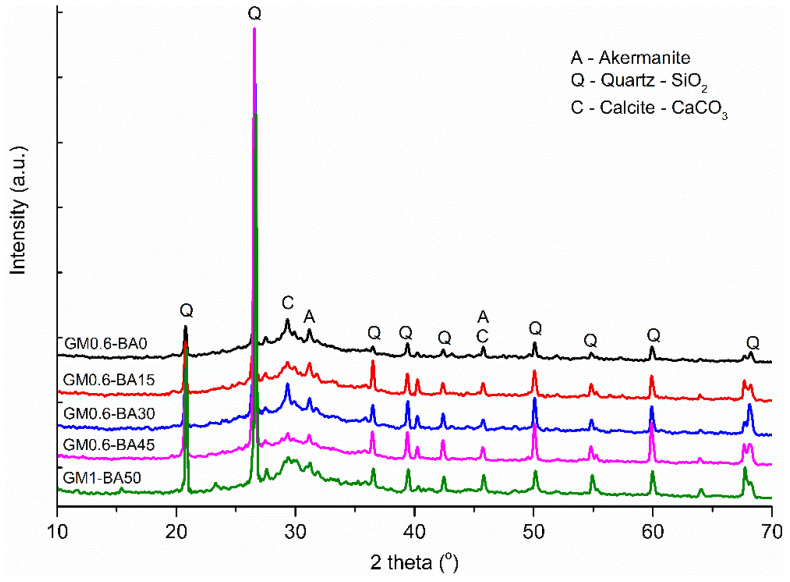
XRD pattern of AA paste samples.

Regarding the compressive strength, Figure 4 illustrates the average of each mix for each curing time (7, 28 and 91 days), corresponding to the average of four tests with their respective standard deviations. In the present research, it is observed that all mix proportions showed the development of compressive strength within the curing time, showing a continuous occurrence of reaction. FTIR (Figure 5) and XRD (Figure 6) analysis suggested that one of these products may be an amorphous gel (CASH and/or NASH), which has strong binder properties. Moreover, the addition of SCBA caused a reduction in the compressive strength cured at the age of 7 days. Besides that, at this age, the compressive strength of GM0.6-BA030 is higher than GM0.6-BA015, which may be related to the SCBA contribution as a silica source for the polymerization increase producing more ion linking and, consequently, strength development. However, at early ages, when there is an excess of silicon in the solution, the reactions are delayed, resulting in a slower strength gain or even no benefit for the material strength [37,38,39]. Note that the increasing SCBA consequently increases the SiO_2_:Al_2_O_3_ ratio and decreases the AA pastes’ compressive strength [40], being more intense in the GM0.6-BA45 sample at 28 days of age (reduction of 52%).

As can be seen, there was an increase of 17% in GM0.6-BA015 at 28 days and 4% in GM0.6-BA030 at the same age, suggesting that the addition of SCBA up to 30% in this type of AA paste is beneficial for mechanical performance. GM0.6-BA015 and GM0.6-BA030 cured at the ages of 28 days reached the highest compressive strength when compared to others at the same age. In addition, GM0.6-BA015 sample reached almost 100 MPa at 28 days, a value that is only reached by GM0.6-BA0 after 91 days of curing. GM0.6-BA45 paste has a slower development, remaining practically stable up to 28 days, followed by a slight growth at 91 days. Although GM0.6-BA45 and GM1-BA50 pastes have reached the lowest strength values in this work, they are still considered high comparing with other studies using SCBA in AAM [11,12]. Furthermore, all tested activated alkaline pastes meet the international standard specifications of 28 MPa for hydraulic cement [41]; moreover, activated alkaline materials need exhibit resistance above 40 MPa to meet the Brazilian standard [42]. Analysis of variance (ANOVA) was performed in order to better understand the results obtained mathematically. Table 1 shows that all variables studied (days of curing, %SCBA and the interaction between them) show significant variation (*p*-value < α (0.05)). Thus, it can be seen that there is a variation in strength in relation to the SCBA content and to the curing days (7, 28 and 91 days).

In the FTIR spectra (Figure 5), it can be seen that the presence of bands between 3200–3700 cm^−1^ and 1630–1640 cm^−1^, attributed to the vibration mode groups H-OH and OH, respectively, suggesting the formation of hydrated products in the samples [43], and to the presence of free water in the sample [44]. The results were obtained after curing 91 days. The band at 775 cm^−1^, present in MK and SCBA, is no longer present in paste sample spectra, which may be related to the dissolution of Si-O bonds present in the amorphous phase, attributed to this band.

It can also be seen that the 900–1200 cm^−1^ band shifted towards lower wave numbers when compared to SCBA and MK. Since the Al-O bond is weaker and longer than the Si-O bond, this shift suggested an elongation of the Si-O-T bond (T: Al or Si) and a reduction in the bond angle, possibly caused by the replacement of Si^4+^ by Al^3+^ ions [26]. The main band in the 960 cm^−1^ region and the bands located in lower wave numbers (600–400 cm^−1^) are associated with the formation of CASH and CSH gels [45], due to asymmetric stretching vibration of Si-O-T bonds in the structure of gel-network [44]. In all AA pastes, the band located in the 1400 cm^−1^ region, typical of the vibration of the elongation of the C-O bond, can be attributed to the presence of carbonates [46], probably due to the presence of CaCO_3_. This suggests that some carbonation process could have occurred in the sample. Based on these, the process and mixtures proposed caused structural changes in the examined AA paste samples, which are attributed to the formation of a new reaction product characteristic of AAM. However, it is noteworthy that the change in the condition of the system directly impacts the reaction rate and the system equilibrium.

The XRD pattern (Figure 6) shows a crystalline phase related to the presence of quartz and calcite. The appearance of a halo between 28–30° in all analyzed samples cured at 28 days could be related to the formation of amorphous gels, such as CASH, CSH and NASH [31,45]. This was also corroborated by FTIR analysis (Figure 5). The presence of quartz is related to unreacted particles present in the precursors, mainly in SCBA and MK, as pointed out in the XRD analyzes of the precursors (Figure 6). In this line, in the alkaline activation of the binary system formed by metakaolin and BFS, dynamics of compensation occur for the formation of the binding gel, so at low alkalinity, the gel formation via MK dissolution is favored, and, on the other hand, when the solution reaches high pH, activation via BFS stands out in relation to MK [46]. Thus, the chemical analyzes suggested that the alkaline activation reaction was effective with the proposed precursors and activators, since the SCBA addition on binary system MK and BFS contributed to the appearance of new reaction products, which could be confirmed by FTIR analysis.

**Figure 7:**
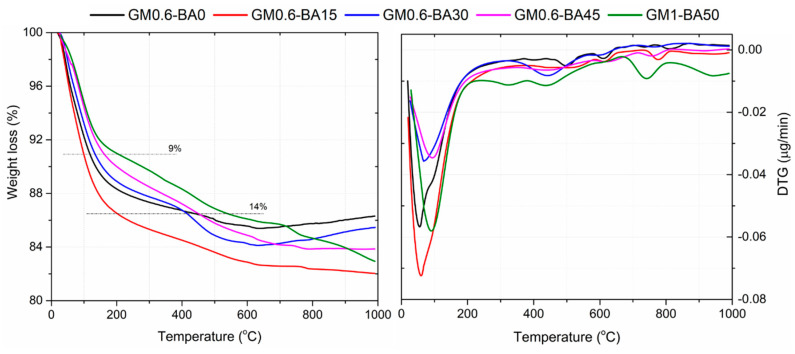
Thermogravimetric analysis of AA paste samples at 91 days.

In the results of thermogravimetry and differential thermogravimetry analysis (TGA/DTG) (Figure 7), the remarkable weight loss occurred between temperatures of 0 and 250 °C, followed by a gradual loss up to a temperature of 1000 °C. The weight loss at 100 °C could be related to the evaporation of free water. The total weight losses in pastes GM0.6-BA0, GM0.6-BA015, GM0.6-BA030, GM0.6-BA45 and GM1-BA50 were 13.70, 17.97, 14.54, 16.15 and 17.07%, respectively. The lower weight loss could be related to a denser microstructures, since looser particles allow free and chemically bound water to be released more easily [47]. The decomposition and dehydration of aluminosilicate gels and organic content was noticed around ~100–200 °C, with the evaporation of water from the system, followed by up to ~600 °C with the release of chemically bound water, continuing at approximately 800 °C [48,49,50]. Among the SBCA pastes, the highest weight loss associated to GM0.6-BA015 could be related to higher aluminosilicate gel formation in this material, while the lowest (GM0.6-BA030 and GM0.6-BA45) could be related to lower formation. Furthermore, decompositions between 400 and 800 °C can be related to carbonates, as calcite, which decompose into CaO and CO_2_, which was more expressive in GM1-BA50 [36,51].

**Figure 8:**
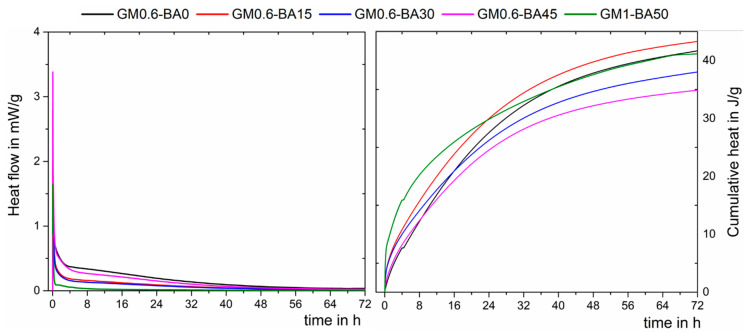
Heat evolution of AA paste samples at 20 °C.

Understanding the evolution of heat provides important information about the processes involved during the reactions. The alkaline activation process involves several steps, which occur both serially and simultaneously [52]. The isothermal calorimetry heat may indicated the formation of reaction products, as shown in Figure 8. Whitin the first hours, the interaction of the precursor particles with the activator releases a significante amount of heat. The total heat released after 72 h was higher for samples GM0.6-BA015 and GM0.6-BA0, followed by GM1-BA50, GM0.6-BA030 and GM0.6-BA45, being 43.3, 41.7, 41.2, 38.3 and 34.8 J/g, respectively. It can be seen that in the cumulative heat, for all pastes, there is a markedrelease of heat up to ~30 h of testing, suggesting that during this period, new reaction products were being produced. Thus, increasing the ash content caused a growing in the cumulative heat, especially in the initial hours ( ~15 h).

The heat flow and cumulative heat (Figure 8) are lower than those reported for cement-based materials [53,54,55], which is commonly over 100 J/g. The low heat released is considered beneficial, as it decreases the potential for thermal cracking and shrinkage [56]. A higher cumulative heat associated with GM0.6-BA15 could be related to the higher aluminosilicate formation, while a lower cumulative heat is associated with GM0.6-BA45 and lower aluminosilicate formation. This result is supported by the compressive strength result. The characteristic of the peaks and the stage (hours) in which they occur vary according to the alkali-activated system studied, and may also presents as two or three exothermic peaks [57,58,59,60]. The single peak shown in Figure 8 could be related to a possible overlapping of the peaks, followed by a progressive release of the reaction products, as pointed out by other authors [61,62]. The occurrence of a single peak is related to the combination of particle wetting steps and a rapid dissolution and precipitation of reaction products [58,59], probably caused by high alkalinity. In this line, the reaction products are continuously formed, as the Ca^2+^ ions are gradually released into solution, since while the solubility of silica and alumina increases with increasing pH, the solubility of calcium decreases [59,63]. The results indicate that there was a gradual formation of products, which provided the development of material strength within curing ages, corroborating compressive strength results (Figure 4). Besides that, among the GM0.6 pastes, the one with lower calcium content and higher silicon content (GM0.6-BA45) shows lower reactivity, which may indicate that these ions directly influence the reactions rate in these materials.

## 3. Materials and Methods

### 3.1. Materials

**Figure 9:**
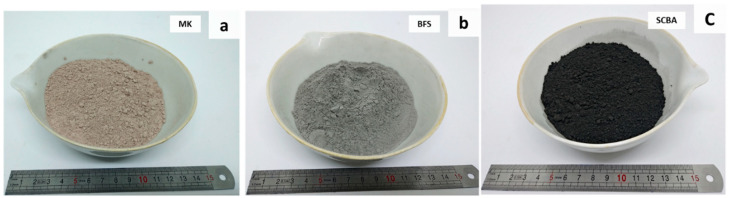
Visual analysis of (**a**) MK, (**b**) BFS and (**c**) SCBA.

Granulated blast furnace slag (BFS), metakaolin (MK) and sugarcane bagasse ash (SCBA) were used as solid precursor in this work to develop AA pastes (Figure 9) samples. MK was supplied by Metacaulim do Brasil, Jundiaí, Brazil, and SCBA from sugar mill BEVAP Bioenergia, João Pinheiro, Brazil. These raw materials were dried in an oven at 100 ± 5 °C for 24 h in order to increase the surface area, and consequently, the chemical reactivity [21]. SCBA exhibits a black color (Figure 9c), which signifies a high unburnt carbon content due to inefficient combustion of biomass [64].

**Figure 10:**
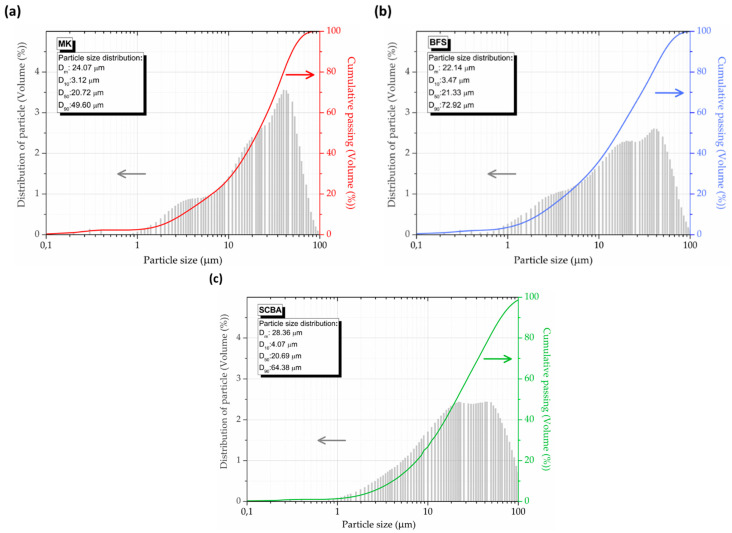
Particle size distribution of the precursors: (**a**) MK, (**b**) BFS and (**c**) SCBA.

The reduction of SCBA particle size contributes to the development of more homogeneous and dense matrices, with better packing of particles and smaller pores [65,66], resulting in a more efficient alkaline activation by providing amorphous silicates to the alkaline solution and the formation of a structured of sodium aluminosilicate hydrate (NASH) or calcium silicate hydrate (CASH)-type network [27]. NASH is formed by precursors with low calcium content, whereas CASH is produced by high-calcium precursors [7]. These geopolymerization products are directly related to the mechanical and durability of AAM [67,68]. Thus, after previous laboratory tests, the ash was ground in a high-performance planetary mill at 300 rpm (in 500 mL pots and 16 zirconia oxide spheres of 10 mm diameter) for 12 min (after time trials of 2, 4, 6, 8, 10 and 12 min). The particle size distribution of precursor was obtained by laser diffraction analysis using CILAS 1090 [69]. The mean particle size (D_m_) of the precursor is around 25 μm, being 24.07, 22.14 and 28.36 μm for MK, BFS and SCBA, respectively (Figure 10).

The chemical oxides in SCBA, MK and BFS (Table 2) were identified by X-Ray fluorescence (XRF) Shimadzu equipment (Tokyo, Japan). The loss on ignition (LOI) value of 11.5% is similar to other research studies that reported values more than 10% for SCBA without further treatment [12,22,26].

**Figure 11:**
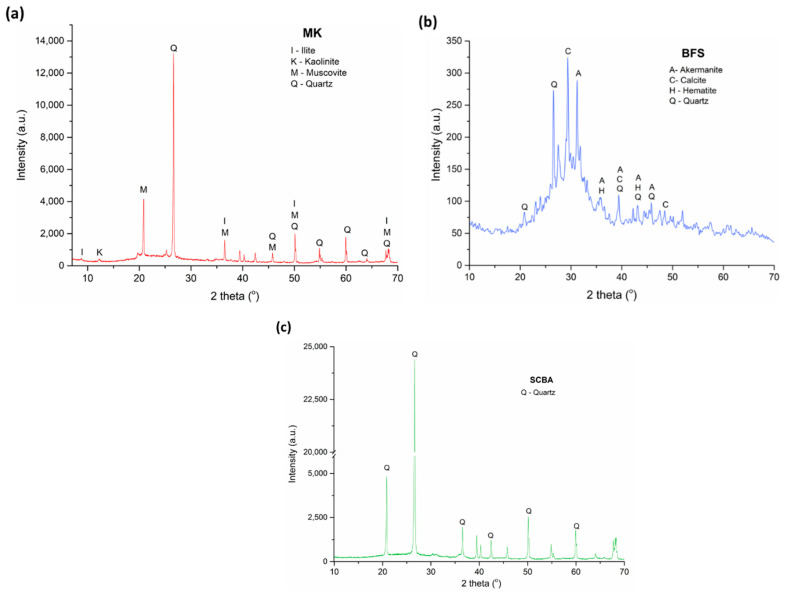
XRD patterns of precursors (**a**) BFS, (**b**) MK and (**c**) SCBA.

The oxide composition showed that silica (SiO_2_) and alumina (Al_2_O_3_) are the main oxides identified in MK and SCBA (Table 2), while BFS also exhibits a high calcium oxide (CaO) and Fe_2_O_3_ content. In this way, the XRD pattern (Figure 11) shows that quartz (SiO_2,_ COD 969013322) is the main crystalline phase in SCBA, and this could be related to sand adhered to the bagasse in the crop field [70]. The baseline of the diffractogram has a deviation, suggesting a proportion of an amorphous phase in the sample. This amorphous hump could be related to amorphous silica present in bagasse composition and the presence of carbon of unburned material [71]. Moreover, the MK diffractogram identified crystalline phases illite (COD 969013719), kaolinite (COD 969009235) and muscovite (COD 969006330). The BFS presented a vitreous phase identified as an amorphous hump in the 20°–35° region [12,31], along with crystalline phases as calcite (COD 969009668), akermanite (COD 969006942) and hematite. The main crystalline phases of MK are muscovite and quartz. In addition, MK exhibits a high content of crystalline phases, which could reduce its reactivity [72].

**Figure 12:**
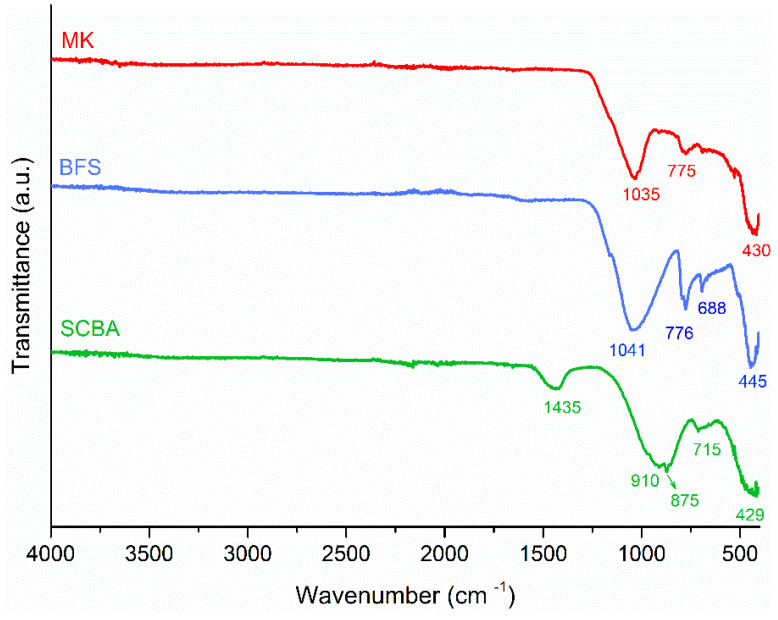
FTIR image spectra of MK, BFS and SCBA.

The FTIR spectra of the precursors (Figure 12) identified wave numbers between 2000 and 400 cm^−1^. The main peak is located in the region of 900–1200 cm^−1^ and it is associated to Si-OT asymmetrical stretching vibrations (T being Si or Al tetrahedral) [73]. In MK, this peak is around 1030 cm^−1^ and is deeper than the peaks in SCBA and BFS, which are located in the 1040 cm^−1^ and 910 cm^−1^ band, respectively. Moreover, in BFS spectra, a shoulder is signed around 870 cm^−1^, which is associated with Al-O asymmetric stretching [74] present in the vitreous phase [43]. Carbonate traces are identified at 1430 and 710 cm^−1^ and are associated with the O-C-O asymmetric elongation mode of the CO_3_^2−^ anion group [75]. In MK and SCBA, the band located at 770 cm^−1^ is associated with the Al-O elongation vibration, and in the regions of 500–400 cm^−1^ with the vibration of the Si-O-Al and Si-O-Si bond [57,70,76] and also with the bending of amorphous silica [26]. The appearance of Si-O bands identified in the SCBA spectrum can be related to the presence of quartz in the material, as also identified by XRD analysis (Figure 11c).

**Figure 13:**
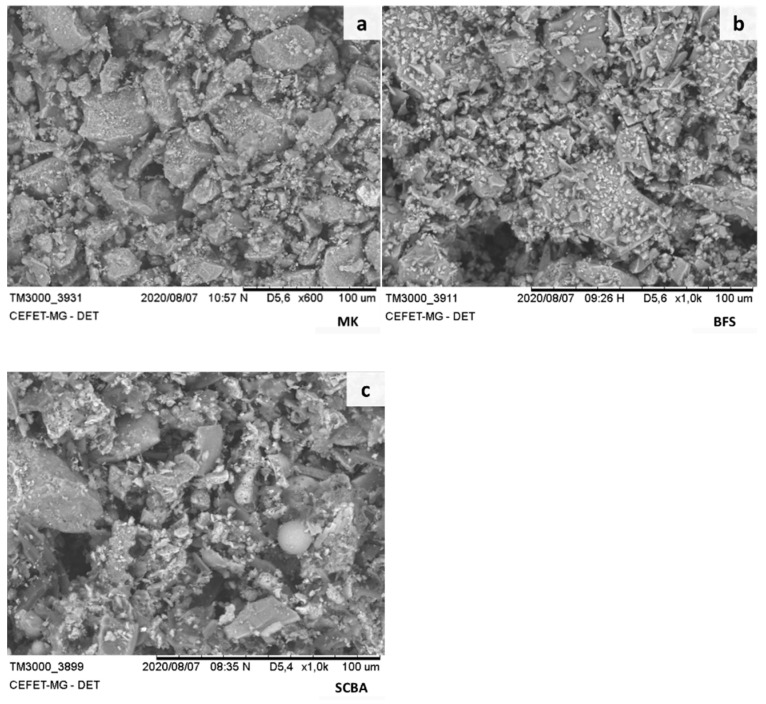
SEM images of the precursors: (**a**) MK, (**b**) BFS and (**c**) SCBA.

Morphology of precursors particles were observed that BFS and MK had particles with different size and shapes (Figure 13a–c). The images were obtained by a scanning electron microscope (SEM) TM-3000 Hitachi equipment (Tokyo, Japan) under low vacuum with a backscattered electron detector and electron acceleration voltage of 15 kV. The presence of sugarcane bagasse fibers (Figure 13c) can be related to a partial or unburnt particle, and it is in accordance with an LOI value of 11.5% (Table 2).

### 3.2. Methods

#### Paste Samples’ Preparation and Characterization

It is well known that BFS and MK are precursors commonly used in AAM; thus, the present work seeks to understand the effect of SCBA incorporation in the MK-BFS system. The alkali-activated paste samples were formulated (Table 3) with varied content of SCBA (45, 30, 15 and 0%) and a constant GBFS:(GBFS + MK) ratio of 0.6. After laboratory tests, the activator:precursor ratio was set at 0.60 and the mixtures were cured at 25 ± 5 °C.

The alkali activator solution was a mixture of sodium hydroxide (NaOH, 8 mol/L) and sodium silicate solution (Na_2_O 14.86%, SiO_2_ 32.07%, H_2_O 52.16%), with a total modulus (SiO_2_/Na_2_O molar ratio) of 1.43, in accordance with the range of values used by other authors in the alkali activation [77] of agro-industrial wastes [11,12,26,47]. Table 3 illustrates the mix proportion and the oxides ratios of all pastes. The SiO_2_:Al_2_O_3_ ratio ranged from 5.26 to 6.85, reaching 10.14 in the GM0-BA50 sample.

A wide range of SiO_2_:Al_2_O_3_ ratios proved to be efficient for alkaline activation [40,78], which coincides with the ratio range found in this work. It can be seen that, due to the high content of SiO_2_ in its composition, increasing the addition of SCBA increases the SiO_2_:Al_2_O_3_ ratio. Furthermore, the CaO content in the system drastically reduces in samples GM0.6-BA45 and GM0-BA50. The GM0-BA50 sample reached a compressive strength average of 1.5 MPa, and also disaggregated easily with handling. Because of this, only the mixture with 50% SCBA–50% BFS (GM1-BA50) continued in this study. This sample fragility was caused by the insufficient chemical interaction between the raw materials, which may be related to the cure temperature, since some systems require thermal curing to develop an early strength [57,79].

Thus, to prepare the pastes, the solid part was mixed in a planetary mixer for 1 min to homogenize the precursors. Then, the activator solution was added and mixed at low speed for 2 min. The fresh pastes were poured into cylindrical metal molds (25 × 50 mm) and vibrated in the vibration table in order to release entrapped air bubbles. The samples were cured at room temperature for 24 h; the surface was covered with glass plates to avoid water evaporation. After this period, the samples were demolded and stored in a closed container until they reached the curing ages of 7, 28 and 91 days.

The crystalline mineralogical characterization of raw materials (MK, SCBA and BFS) and pastes was obtained by X-Ray diffraction (XRD) Shimadzu equipment (Tokyo, Japan) using Cu-kα (λ = 1.5418 Å), at 40 kV and 30 mA, and an angle measurement interval (2θ) between 10°and 80° and step size of 0.02; for phase identification, the Match! 3 software was used [80] with the Crystallography Open Database (COD) revision no. 254652. FTIR, compressive strength, SEM analyses, water absorption and calorimetry techniques were performed in the pastes after curing time. Fourier Transform Infrared Spectroscopy (FTIR) analysis was performed with IR-Prestige-21 Shimadzu equipment; the spectrum wavelengths ranged between 4000 cm^−1^ and 400 cm^−1^ [7,69]. Moreover, the microscopy experiments of paste samples were performed on a scanning electron microscopy (SEM) model Hitachi TM-3000 (Tokyo, Japan) under low vacuum with a backscattered electron detector and electron acceleration voltage of 15 kV.

Thermogravimetric analysis (TGA) Hitachi equipment (Tokyo, Japan) was performed with the temperature range of 25 °C–1000 °C at 10 °C/min in a nitrogen environment at 60 mL/min purge rate in a STA7300 instrument. Samples were crushed, transferred immediately to an alumina crucible and held under isothermal conditions. The hydration heat evolution of paste samples was assessed by isothermal calorimetry, I-calorimeter Cal-4000 4 channel Calmetrix equipment (Arlington, MA, USA), at a temperature of 23 °C, for 72 h of reaction. In this analysis, fresh paste was mixed externally and immediately placed in the calorimeter. The samples were composed of 15 g of precursors and 9 g of activators, keeping the activators:precursors ratio 0.6 for all tests. For the mechanical characterization, a compressive strength test was conducted according to Brazilian standard NBR 7215 [81] at curing ages of 7, 28 and 91 days, with an increasing stress rate of 0.25 MPa/s in an EMIC DL 30000 universal test machine (São José dos Pinhais, Brazil), and the programs TESC and Vmaq were used. Variance analysis (ANOVA) of the compressive strength results were conducted using the software package Minitab 17 to validate the results. The water absorption, apparent porosity, bulk density (dry and saturated) and apparent density were performed and calculated based on Brazilian standard NBR 9778 [82].

## 4. Conclusions

This work studies the feasibility of using sugarcane bagasse ash as a precursor in alkali-activated pastes in a ternary system, with metakaolin and granulated blast furnace slag. The conclusions drawn from the physical, chemical, and mechanical characterizations obtained in this study are as follows:

- SCBA can be used as a precursor in alkali-activated pastes in a ternary cement with a metakaolin and granulated blast furnace slag (BFS/(BFS + MK) ratio of 0.6. The high SiO_2_/Al_2_O_3_ ratio and presence of crystalline particles contributed to the development of AA pastes;
- XRD analysis revealed the appearance of a typical hump around 28–30 in all samples cured at 28 days, suggesting the formation of a typical AAM gel. This statement is also confirmed by other analyses, such as FTIR, TGA and SEM;
- The addition of SCBA had an important role in GM0.6-BA015 and GM0.6-BA030, increasing the compressive strength without causing a significant effect on water absorption, porosity and bulk density;
- The increase in the SCBA content causes a reduction in strength and a considerable increase in water absorption, as occurred with samples GM0.6-BA45 and GM1-BA50. However, compared with other works using OPC and international standards, these samples showed expressive strength.

Research such as this allows the options to expand the possibilities for precursors in AAM. This enables, therefore, implementation of SCBA as precursor according to regional availability, leading to environmental benefits and adding value to waste that is still deposited in landfills. However, it is noteworthy that there are still obstacles to overcome regarding the practical applicability of the large-scale use of SCBA in building materials, such as the variability in geographic distribution, transport and logistics costs and diversity of materials processing in sugarcane mills, among others. Therefore, further research on processing and parameter optimization for the application in AAM is highly recommended.

## Figures and Tables

**Table 1:** Analysis of variance (ANOVA) for the proposed samples and curing time.

Source of Variability	Sum of Squares	F Value	*p* Value
Days of curing	12,404.1	292.09	0.000
%SCBA	12,434.6	195.20	0.000
%SCBA days of curing	5165.0	40.54	0.000
Error	764.4		
Total	30,768.0		

**Table 2:** Chemical composition of precursors metakaolin (MK), slag (BFS) and sugarcane bagasse ash (SCBA) (mass%).

	SiO_2_	Al_2_O_3_	CaO	Fe_2_O_3_	MgO	K_2_O	Na_2_O	TiO_2_	P_2_O_5_	LOI
MK	60.0	32.5	0.1	1.8	0.2	1.6	0.1	0.9	-	2.6
BFS	35.3	5.4	45.8	24.3	3.0	-	-	-	-	-
SCBA	65.8	13.6	2.3	4.5	1.3	2.0	-	0.9	0.9	11.5

**Table 3:** Mix proportion and oxide ratio of the samples.

Sample	GBFS/(GBFS + MK)	SCBA (%)	SiO_2_: Al_2_O_3_	CaO: SiO_2_
GM0.6-BA0	0.6	0	5.26	0.55
GM0.6-BA15	0.6	15	5.77	0.45
GM0.6-BA30	0.6	30	6.60	0.40
GM0.6-BA45	0.6	45	6.85	0.28
GM1-BA50	1	50	10.14	0.45
GM0-BA50	0 *	50	5.10	0.02

* Note: 0% GBFS and 50% MK.

## Data Availability

The data presented in this study are available on request from the corresponding author.

## Sample Availability

Samples of the compounds presented in the paper are available from the authors.
